# Testis-Specific Thioredoxins TXNDC2, TXNDC3, and TXNDC6 Are Expressed in Both Testicular and Systemic DLBCL and Correlate with Clinical Disease Presentation

**DOI:** 10.1155/2021/8026941

**Published:** 2021-02-01

**Authors:** Mikko C. Chan, Janette Savela, Riina K. Ollikainen, Hanna-Riikka Teppo, Ilkka Miinalainen, Risto Pirinen, Esa J. M. Kari, Hanne Kuitunen, Taina Turpeenniemi-Hujanen, Outi Kuittinen, Milla E. L. Kuusisto

**Affiliations:** ^1^Cancer and Translational Medicine Research Unit, University of Oulu, Finland; ^2^Department of Oncology and Radiotherapy and Medical Research Center, Oulu University Hospital, Oulu, Finland; ^3^Department of Pathology, Oulu University Hospital, Oulu, Finland; ^4^Biocenter, University of Oulu, Oulu, Finland; ^5^Department of Pathology, North Karelia Central Hospital, Joensuu, Finland; ^6^Department of Oncology, Faculty of Health Medicine, Institute of Clinical Medicine, University of Eastern Finland, Kuopio, Finland; ^7^Department of Hematology, Oulu University Hospital, Oulu, Finland

## Abstract

DLBCL is the most common type of non-Hodgkin lymphoma with a substantial group of patients suffering a poor prognosis. Therefore more specific markers are required for better understanding of disease biology and treatment. This study demonstrates that testis-specific antioxidant enzymes TXNDC2, TXNDC3, and TXNDC6 alongside oxidative stress marker 8-OHdG are expressed in both testicular and systemic DLBCL, and their presence or absence has correlations with clinical risk factors such as the number of extranodal effusion, the appearance of B-symptoms, and treatment response. Biopsy samples were collected from 28 systemic and 21 testicular male DLBCL patients. The samples were histostained with TXNDC2, TXNDC3, TXNDC6, and 8-OHdG, then graded by a hematopathologist blinded to clinical data. Immunoelectron microscopy was used as a second method to confirm the reliability of the acquired immunohistochemistry data. The absence of nuclear TXNDC2 expression in testicular DLBCL cells correlated with worse primary treatment response, cytoplasmic TXNDC3 expression in testicular and systemic DLBCL associated with lower frequency of B-symptoms, and TXNDC6 expression in cytoplasm in systemic DLBCL had a clinical significance with higher LD levels suggesting a role in the biological nature of these lymphomas. Overall, TXNDC3 cytoplasmic expression is correlated with a more positive outcome in both testicular and systemic DLBCL, while TXNDC6 cytoplasmic expression is associated with a negative outcome in systemic DLBCL.

## 1. Introduction

Diffuse large B-cell lymphoma (DLBCL) is the most common type of non-Hodgkin lymphoma (NHL). Over half of patients respond well to current immunochemotherapy regimens, whereas the prognosis of the chemorefractory and relapsed disease is poor [1]. The second-line therapies have response rates (RR) ranging from 1% to 14% with median overall survival less than 5 months [2]. One factor that explains poor prognoses in these cases is the involvement of DLBCL in the central nervous system (CNS). About 5% of DLBCL patients will have CNS relapse [3]. NHL can also manifest in the CNS system only, when it is called as primary CNS lymphoma (PCNSL). Most of these lymphomas represent with DLBCL histology. PCNSL and primary testicular DLBCL belong to heterogeneous groups of lymphomas, which are both immunoprivileged site-associated DLBCLs [4]. The mechanism of how these lymphomas develop to immunoprivileged sites is currently not well known.

Reactive oxygen species (ROS) are produced continuously in aerobic conditions, and the intracellular redox environment is dependent on the production and elimination of ROS. Antioxidant enzymes scavenge ROS, and oxidative stress develops when the amount of ROS exceeds the rate of removal. This may damage DNA and lead the cell to apoptosis [5]. 8-Hydroxydeoxyguanosine (8-OHdG) is an oxidative stress marker caused by hydroxyl radical, and it is used as a surrogate molecular marker of oxidative stress [6]. Alongside with 8-OHdG, glutathione peroxidase 4 (GPX4) can act as an independent prognostic factor in DLBCL. GPX4 is an antioxidant enzyme that has a role in the ROS balance in which the overexpression of GPX4 suggested worse prognosis [7].

Thioredoxins (Trx) can be found in all living cells, and they take part in oxygen metabolism protecting against oxidative stress damage. They are linked to many human diseases, including cancers [8]. The Trx family has a major role in maintaining the redox state inside cells [9]. The family is divided into two groups, both sharing a thioredoxin domain. Group I includes Trx-1 and Trx-2, and group II contains, for example, thioredoxin domain-containing (TXNDC) proteins 2, 3, and 6 [10]. They are mainly considered testis-specific proteins. TXNDC2 is mainly found in the tail of elongating spermatids and spermatozoa [11], while TXNDC3 is mainly expressed at the end of spermiogenesis [12]. The highest levels of TXNDC6 have been found in testis and lung and associated with microtubule structures [13].

In this study, we wanted to explore the expression and clinicopathological impact of 8-OHdG, Trx-1, and thioredoxin domain-containing proteins TXNDC2, TXNDC3, and TXNDC6 in testicular and systemic DLBCL patient biopsy samples.

## 2. Materials and Methods

### 2.1. Patient Material

The material consisted of 49 male patients with histologically confirmed *de novo* systemic (*n* = 28) or testicular DLBCL (*n* = 21) and 24 patients with normal testis tissue as controls. The mean age of healthy control patients was 82, and the biopsies were obtained due to the following reasons: orchiectomy as a palliative treatment for prostate adenocarcinoma (13 out of 24), exploratory surgery for undefined testicle pain (6/24), and exploratory surgery for tumor found in testicles (3/24). The detailed disease information of 2 out of 24 patients was not available. Lymphomas were diagnosed and treated at Oulu University Hospital and North Karelia Central Hospital between 1984 and 2014. The mean age of patients with systemic DLBCL was 58.8 and those with testicular lymphoma 76.6. Diagnoses were reviewed by an experienced hematopathologist. The diagnostic workup included medical history and physical examination, blood chemistry, bone marrow aspiration and biopsy, and whole-body computed tomography. Modern immunochemotherapy regimens were used to treat the patients, including CHOP (cyclophosphamide, doxorubicin, vincristine, and prednisolone), with or without rituximab. Patients with testicular lymphoma received radiotherapy to the contralateral testicle (*n* = 7) and regional systemic area (*n* = 3) (Table 1). The study was carried out following the ethical code of the Helsinki Declaration and approved by the Regional Ethics Committee of Northern Ostrobothnia Hospital District (42/2010) and by the National Supervisory Authority for Welfare and Health Valvira (9580/05.01.00.06/2010).

### 2.2. Immunohistochemistry

Diagnostic surgical biopsies were removed via hospital diagnostic procedures and were collected from hospital archives. Sections of 5 *𝜇*m thickness were cut from samples routinely fixed in formalin and embedded in paraffin and placed on Superfrost Plus glass slides (Menzel-Gläser, Braunschweig, Germany). The slides were incubated at +37°C for 4 hours. Then, the slides were deparaffinated in a clearing agent, Histo-Clear (National Diagnostics, Atlanta, GA, USA), and rehydrated in a graded series of alcohol solutions. To retrieve the epitopes, the slides were microwaved for 10 to 15 minutes in Tris-EDTA or citrate buffer (Table 2). The cooling of the slides at room temperature took 20 minutes. After cooling, the slides were incubated in a 3% H_2_O_2_ solution for 5 minutes for the blocking of endogenous peroxidase activity. After that, the slides were incubated with primary antibody in a humidity chamber. Then, the immunostaining was continued using a Dako REAL™ EnVision™ Detection System (Dako Denmark A/S, Glostrup, DK) according to the instructions of the manufacturer. The immunoreaction was detected with diaminobenzidine. PBS-Tween was used to wash the slides between every stage of the immunostaining procedure. In the end, the slides were counterstained with Mayer's hematoxylin (Reagena, Toivola, Finland), dehydrated, and mounted with Histomount (National Diagnostics, Atlanta, GA, USA).

The stainings were reviewed and results analyzed on a multihead microscope by an experienced hematopathologist (HRT) with investigators (JS, RKO) blinded to clinical data. The staining was divided into three groups according to intensity: negative, weak, and strong; the weak intensity was seen only with 40x magnification, whereas the strong intensity could be seen with 10x magnification. Their respective proportions in the cancerous cells were assessed in percentages (100% as total). Then, a modified Histoscore (H-score) was used for statistical analysis: H − score = 0x negative expression (%of cells) + 1x weak expression + 3x strong expression (range 0–300). [14]

### 2.3. Cell Culture

Western blotting was used to examine the expression of TXNDC2, TXNDC3, and TXNDC6 in systemic DLBCL. The commercial cells Farage (ATCC® CRL-2630™) and Pfeiffer (ATCC®CRL-2632™) were purchased from ATCC (ATCC bioproduction facilities, Manassas, Virginia, USA), OCI-LY1 (ACC 772) was purchased from DSMZ (DSMZ-German Collection of Micro-organisms and Cell Cultures GmbH, Braunschweig, Germany), and Karpas 422 (ECACC 06101702) was purchased from ECACC (European Collection of Authenticated Cell Cultures, Salisbury, UK) and cultured in cell culture bottles for suspension cells. The medium used for Farage and Pfeiffer was RPMI-1640 which included 10% FBS, Penicillin Streptomycin, 1 mM Sodium Pyruvate, and 10 mM HEPES, and for Karpas 422, the FBS was 20%. IMDM (1x) medium which included 20% FBS, Penicillin Streptomycin, 1 mM Sodium Pyruvate, and 10 mM HEPES was used for OCI-LY1 cells. All reagents were from HyClone (GE Healthcare Life Sciences, HyClone Laboratories, Utah, USA).

Subcellular fractioning was performed using the ThermoFisher Scientific Subcellular Protein Fractionation Kit for Cultured Cells-78840 according to the manufacturer's instructions. After this, the concentrations of proteins were measured with Bio-Rad DC Protein Assay (Bio-Rad Laboratories, Hercules, CA, USA). When concentrations were equalized, the Laemmli buffer was added. Then, the samples were boiled and stored at -80°C.

### 2.4. Western Blotting

The same amounts of protein samples were separated on SDS-PAGE. The proteins were transferred to PVDF membranes, and after this, the membranes were blocked with 5% BSA (1x PBS, 0.1% Tween-20, 0.005% sodium azide) and incubated in primary antibodies overnight at +4°C. The horseradish peroxidase- (HRP-) linked secondary antibody was used (Table 2), and the membranes were developed using the chemiluminescence. After this, the membranes were exposed to radiographic film.

### 2.5. Immunoelectron Microscopy (IEM)

For IEM analysis, the biopsy sample used was from the systemic DLBCL IHC series as described previously. The sample was fixed in 4% paraformaldehyde in 0.1 M phosphate buffer (pH 7.4) with 2.5% sucrose for 2 hours. Tissue pieces were immersed in 2.3 M sucrose in PBS and rotated at +4°C for 4 hours. Specimens were frozen in liquid nitrogen, and thin cryosections were cut with Leica EM UC7 cryoultramicrotome (Leica Microsystems, Vienna, Austria). The sections were picked on Butvar-coated nickel grids. The grids were first incubated in 2% gelatin in PBS for 20 minutes and then in 0.1% glycin-PBS for 10 min followed by incubation in a blocking serum containing 1% BSA in PBS for 5 minutes. 1% BSA in PBS was used in washings and dilutions of antibody and gold conjugates. Sections were exposed to the primary antibodies (Novus Biologicals, CO, USA) against TXNDC-2, TXNDC-3, and TXNDC-6 for 60 minutes, followed by incubation with protein A conjugated 10 nm gold (Cell Microscopy Core, University Medical Center Utrecht, Netherlands) for 30 minutes. The controls were prepared by replacing the primary antibody with PBS. The grids were stained with neutral uranyl acetate (UA) and embedded in 2% methyl cellulose containing 0.4% UA and examined with a Tecnai Spirit transmission electron microscope (FEI, Eindhoven, Netherlands). Images were captured by a Quemesa CCD camera (Olympus Soft Imaging Solutions GMBH, Munster, Germany).

### 2.6. Statistical Analysis

IBM SPSS Statistics 24.0 for Windows (IBM Corporation, Armonk, NY, USA) was used for statistical analysis. Nominal variables (such as the clinical determinants) were evaluated with the chi-square test and with Fisher's exact test. Continuous variables (TXNDC markers and 8-OHdG) were tested with the Mann–Whitney *U* test, Levene's test, and the Kruskal-Wallis test, and in determining a two-classed variable for survival analysis, Histoscore cut-off values were chosen for TXNDC and 8-OHdG using a receiver operating characteristic curve (ROC). The Kaplan-Meier estimator was used to observe the correlation with survival variables OS, disease-specific survival (DSS), and progression-free survival (PFS). *p* values < 0.05 were considered statistically significant.

## 3. Results

### 3.1. 8-Hydroxy-2′-deoxyguanosine

**Figure 1:**
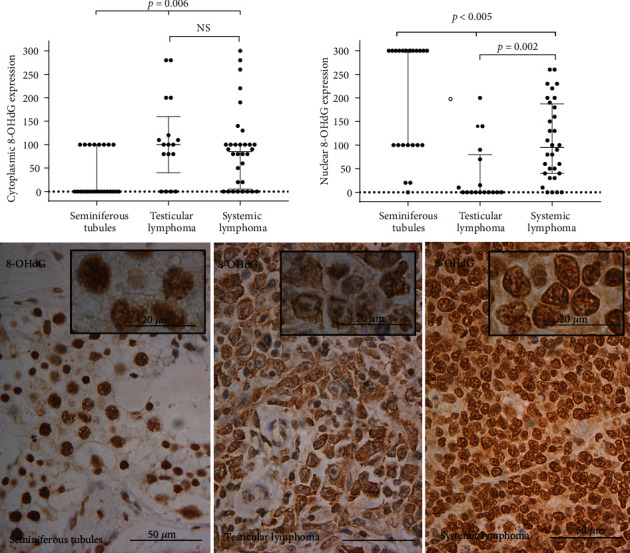
The expression of 8-OHdG in the patient sample set. Dot plots represent the Histoscore (range 0-300), and micrographs represent the average expression of 8-OHdG in the diagnostic groups. Kruskal-Wallis test was used to calculate the *p* value. NS: not significant.

Immunoreaction for cytoplasmic 8-OHdG expression remained mostly absent (16 out of 24) and only partly weakly positive in normal testis tissue samples (Figure 1, dot plot). Nuclear 8-OHdG expression was positive in 23 out of 24 samples with a median modified Histoscore value of 300. Cytoplasmic expression of 8-OHdG in testicular lymphoma samples had a median modified Histoscore value of 85 when the nuclear 8-OHdG expression seemed to be absent. Both cytoplasmic and nuclear 8-OHdG expression was seen in systemic lymphoma samples with median modified Histoscore values of 85 and 95, respectively.

Cytoplasmic 8-OHdG expression was lower in normal testis tissue samples compared to testicular and systemic lymphoma samples (*p* = 0.006), but the difference between testicular and systemic lymphoma samples was insignificant (Figure 1, dot blot). The expression of nuclear 8-OHdG in normal testis samples was higher compared to systemic and testicular lymphoma (*p* < 0.005) and higher in systemic lymphoma compared to testicular lymphoma (*p* = 0.002).

The cytoplasmic expression of 8-OHdG in testicular lymphoma seemed to be associated with the stage (*p* = 0.052), extranodal effusion (*p* = 0.050), and mortality (*p* = 0.056) but showed a distinct correlation with the primary treatment response (*p* = 0.012). In systemic lymphoma, it had, however, no correlations with the observed variables. The absence of immunoreaction of 8-OHdG in the nucleus is correlated with extranodal metastasis (*p* = 0.006) in testicular lymphoma, whereas the positive expression in systemic lymphoma is correlated with the stage of lymphoma (*p* = 0.024) and relapse (*p* = 0.046). 8-OHdG did not have any association with survival variables in these lymphomas.

### 3.2. Thioredoxin Domain-Containing Protein 2

**Figure 2:**
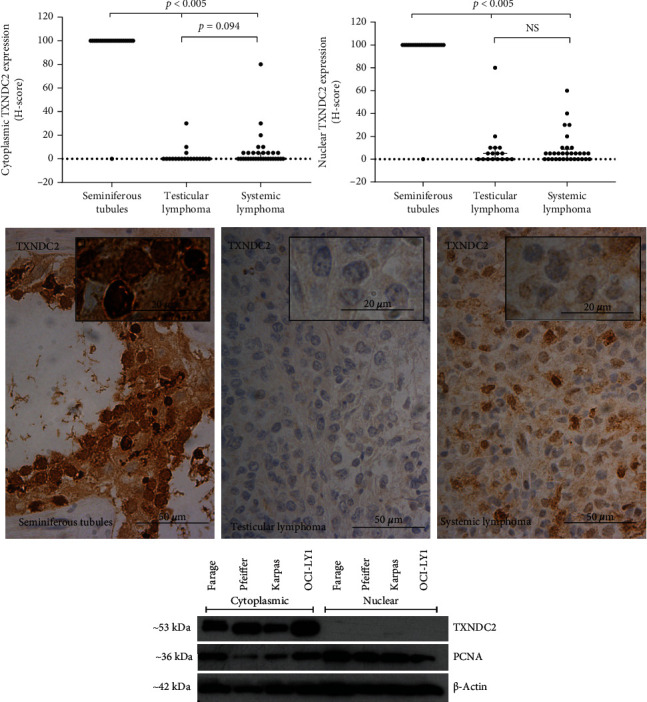
The expression of TXNDC2 in the patient sample set and in the cell culture model. Dot plots represent the Histoscore (range 0-300), and micrographs represent the average expression of TXNDC2 in the diagnostic groups. Western blot with three biological replicates of two different DLBCL cell lines shows that TXNDC2 is expressed in these cell lines representing systemic lymphomas. Kruskal-Wallis test was used to calculate the *p* value. NS: not significant.

TXNDC2 was detected both in the cytoplasm and the nucleus of normal testis tissue samples and weakly in the nucleus of lymphoma samples (Figure 2). Immunoreaction was detected as a weak cytoplasmic and nuclear positivity in all but one normal testis tissue sample (23 out of 24), with a median of 100 in the modified Histoscore.

The majority of the lymphoma samples remained negative in cytoplasm, and this was observed in 19 (59.4%) and 14 (82.4%) samples of systemic and testicular lymphoma, respectively. Nuclear negativity was seen in 13 (40.6%) and 8 (47.1%) samples of systemic and testicular lymphoma, respectively, and the median expression level was 5 in both diagnostic groups in the modified Histoscore. These results are consistent with the experiment where we tested whether the lymphoma cells would able to express TXNDC2 in cell culture. Western blotting, from whole cell lysates of the cell lines representing systemic lymphomas, shows that TXNDC2 can be expressed in systemic DLBCL (Figure 2, Western blot).

The score of the cytoplasmic and nuclear TXNDC2 in normal seminiferous tubule cells was significantly higher than that in systemic and testicular lymphoma (*p* < 0.005, dot blots), but the expression did not vary significantly between the lymphoma groups (Figure 2, dot blots).

In systemic DLBCL, the absence of nuclear expression of TXNDC2 is correlated with one or no extranodal effusion at all (*p* = 0.044), whereas nuclear negativity in testicular DLBCL is associated with worse primary treatment response (*p* = 0.044). There were no associations with survival.

### 3.3. Thioredoxin Domain-Containing Protein 3

TXNDC3 showed strong cytoplasmic positivity in 18 (75%) normal testis tissue samples, whereas 6 (25%) cases were negative, and nuclear expression remained as negative.

**Figure 3:**
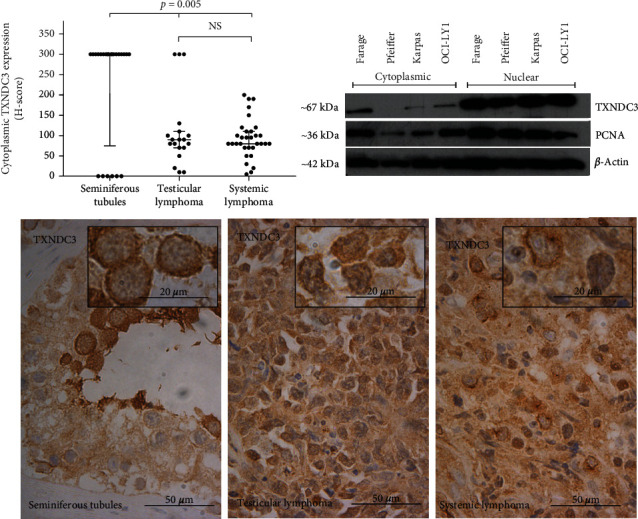
The expression of TXNDC3 in the patient sample set and in the cell culture model. Dot plots represent the Histoscore (range 0-300) and micrographs represent the average expression of TXNDC3 in the diagnostic groups. Western blot with three biological replicates of two different DLBCL cell lines shows that TXNDC3 is expressed in these systemic lymphoma cell lines. Kruskal-Wallis test was used to calculate the *p* value. NS: not significant; H-score: Histoscore.

In all the lymphoma samples, TXNDC3 was expressed in the cytoplasm, and the median scores for the expression were 80 and 90 in systemic and testicular lymphoma, respectively. The nuclei were negative in TXNDC3 staining in all lymphoma samples. Control Western blotting in cell lines shows that TXNDC3 is expressed in systemic DLBCL (Figure 3, Western blot).

The score for the expression of cytoplasmic TXNDC3 in normal seminiferous tubule cells was significantly higher than in systemic and testicular lymphoma (*p* = 0.005, dot blots), but the expression level did not vary significantly between these lymphoma groups (Figure 3, dot blots). 31% of systemic DLBCL samples showed a dot-like expression.

In testicular DLBCL when taking account of only the weak intensity group of TXNDC3 cytoplasmic expression (described in the method section), statistical correlations were found with less B-symptoms (*p* = 0.016), but there was no association with overall Histoscore. In systemic DLBCL samples, cytoplasmic TXNDC3 expression is correlated with less B-symptoms (*p* = 0.017), and when only taking account of the strong intensity group TXNDC3 cytoplasmic expression, association with one or no extranodal effusion (*p* = 0.037) and stage ≤ III (*p* = 0.043) was found. Survival associations were not detected.

### 3.4. Thioredoxin Domain-Containing Protein 6

TXNDC6 showed a weak cytoplasmic positivity in all but one normal testis tissue samples (23 out of 24). There was no detectable nuclear immunoreactivity.

**Figure 4:**
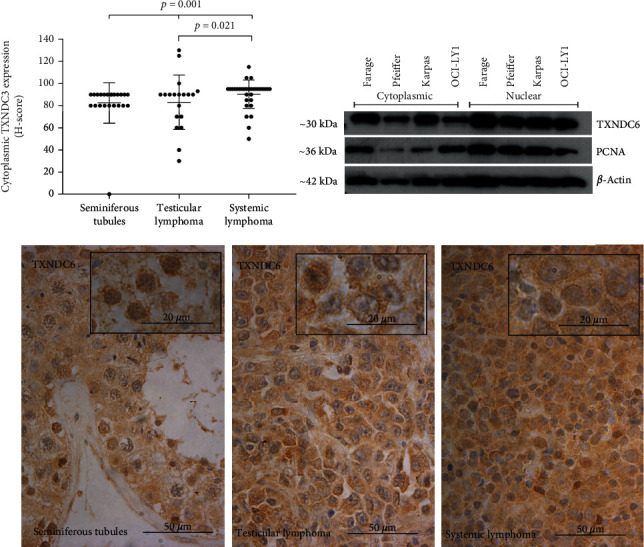
The expression of TXNDC6 in the patient sample set and in the cell culture model. Box plots represent the Histoscore (range 0-300), and micrographs represent the average expression of TXNDC6 in the diagnostic groups. Western blot with three biological replicates of two different DLBCL cell lines shows that TXNDC6 is expressed in these systemic lymphoma cell lines. Kruskal-Wallis test was used to calculate the *p* value. NS: not significant; H-score: Histoscore

A cytoplasmic expression was detected in all of the lymphoma samples, and the median expression Histoscores were 95 and 90 in systemic and testicular lymphomas, respectively. The nuclei were negative in all lymphoma samples. Western blotting, prepared as a control experiment, showed that TXNDC6 can be expressed in these cell lines representing systemic DLBCL (Figure 4, Western blot).

Cytoplasmic expression in normal seminiferous tubule cells was significantly lower than in DLBCL and testicular lymphoma (*p* = 0.001, dot blots), and the expression was significantly higher in systemic DLBCL compared to testicular lymphoma (*p* = 0.021, Figure 4).

No clinical correlations with TXNDC6 expression were detected in testicular DLBCL samples. In systemic DLBCL samples, cytoplasmic TXNDC6 expression correlated with higher LD level (*p* = 0.029), and when taking into account the weak intensity group of cytoplasmic expression, it correlated with lower WHO-class (*p* = 0.029). Survival correlations were, however, not detected.

### 3.5. Immunoelectron Microscopy

**Figure 5:**
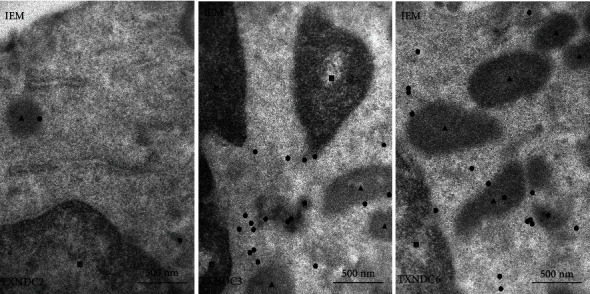
Immunoelectron microscopy (IEM) showing the localization of the expression of TXNDC2, TXNDC3, and TXNDC6 which are marked as circles. Triangles represent mitochondria and squares nuclei.

TXNDC2 was expressed weakly in the cytoplasm and appeared as negative in the nucleus. TXNDC3 expression was not found in the nucleus but was moderately settled near the nucleus in cytoplasm. Nucleus appeared as negative for the TXNDC6 expression as well, and in cytoplasm, the expression was concentrated near mitochondria. These findings confirmed the reliability of the immunohistochemistry used in this study (Figure 5).

## 4. Discussion

Firstly, we showed that the studied lymphomas express the molecular marker 8-OHdG indicating the presence of oxidative DNA damage. The expression level of nuclear 8-OHdG varies greatly in the studied cases of testicular and systemic lymphoma indicating a heterogenous REDOX status. The expression pattern of 8-OHdG in the seminiferous tubules is consistent with the findings in previous publications [15]. Our earlier studies demonstrate that 8-OHdG is associated with aggressive disease presentation in DLBCL [16]. In contrast to systemic lymphomas, the absence of nuclear 8-OHdG in testicular lymphoma was associated with the presence of extranodal effusion; moreover, the presence of cytoplasmic 8-OHdG expression was associated with primary treatment response in testicular DLBCL. To our knowledge, this is the first study to demonstrate the immunohistochemical expression of 8-OHdG in testicular lymphoma.

Antioxidative enzymes protect both normal and malignant cells from oxidative damage; however, excess expression of these enzymes may give cancer cells a survival advantage by inducing proliferation and replicative immortality. Antioxidative enzymes may protect cells from chemotherapeutics [17] such as doxorubicin, a cancer therapeutic drug used in routine DLBCL regimens. Doxorubicin causes the death of cancer cells by topoisomerase II-mediated DNA damage [18], but it also induces ROS formation inside mitochondria and causes ROS overproduction leading, for example, to cardiotoxicity [19]. Trx-1 has been associated with poor prognosis in patients with DLBCL [16], partly because it inhibits the cytotoxicity of doxorubicin.

Unlike Trx-1, Thioredoxin family group II members TXNDC2, TXNDC3, and TXNDC6 are poorly studied in malignancies but described only in normal tissues. TXNDC2 has been seen in the developing tail of the elongating spermatids but not in any tail structure after its completion [11]. TXNDC3 is a sperm fibrous sheath protein and expressed at the end of spermiogenesis in mature spermatozoa but not in the remainder of testicular cells [10, 12]. Functional disruption of TXNDC2 and TXNDC3 leads to an age-dependent loss of sperm motility and an increase of intracellular superoxide anion. Of these TXNDC enzymes, TXNDC6 has been also previously shown to be expressed in the lungs [13].

The limited data concerning their expression and biological role in malignancies shows that TXNDC6 expression is increased in human colon cancer tissues compared to nontumorous tissues and correlates with cancer cell differentiation and disease stage. It is also proven that knockdown of TXNDC6 inhibits motility, invasion, and *in vivo* metastatic properties of colon cancer cells [20]. Our results demonstrate that TXNDC2, TXNDC3, and TXNDC6 are all expressed in testicular and systemic lymphomas. The expression of TXNDCs in cell lysates supports this finding, and the antigen localization determined with immunohistochemistry was further confirmed with IEM. Overall, the expression of TXNDC2 and TXNDC3 was weaker in lymphoma tissue compared to seminiferous tubule cells, whereas the expression level of TXNDC6 was deemed low in all the sample types. Advanced cancer features such as stage were associated with low expression of TXNDC3 in systemic DLBCL, and the low expression of TXNDC3 also correlated with one or more extranodal effusion in both systemic and testicular DLBCL.

However, the number of cases is limited, and this should be regarded merely as a pilot study. The causality of these findings and the mechanism of their actions as well as their impact on treatment resistance warrants further studies. Our results imply that malignancies are able to adopt aberrant antioxidative defense mechanisms not normally expressed in corresponding tissues. Their expression has clinical relevance, and they should also be taken into account when evaluating tumors' antioxidant defense mechanisms and their clinical role.

## 5. Conclusions

This is the first study to demonstrate that testis-specific antioxidant enzymes TXNDC2, TXNDC3, and TXNDC6 are expressed on both testicular and systemic DLBCL and that their presence or absence is associated with several clinical features—for example, the presence of one or more extranodal effusion or the treatment response.

## Figures and Tables

**Table 1:** Demographics of 28 males with systemic diffuse large B-cell lymphoma (DLBCL) and 21 testicular DLBCL patients. The mean age of all patients was 65.0, range 26–90. Correlation between systemic DLBCL and testicular DLBCL was calculated using Fisher's exact test for B-symptoms, age, LD levels, WHO, extranodal effusion, and IPI, whereas correlation with stage was calculated with Pearson chi-square. IPI: International Prognostic Index; LD: lactate dehydrogenase; NA: not available.

	Systemic DLBCL (*n* = 28) *n* (%)	Testis DLBCL (*n* = 21) *n* (%)	*p* value
B-symptoms			0.362
None	12 (42.9)	15 (71.4)	
Yes	14 (50.0)	5 (23.8)	
NA	2 (7.1)	1 (4.8)	
Age			0.001
<60 years	15 (53.6)	1 (4.8)	
≥60 years	13 (46.4)	20 (95.2)	
LD levels			0.320
Normal	10 (35.7)	10 (47.6)	
Elevated	16 (57.2)	6 (28.6)	
NA	2 (7.1)	5 (23.8)	
Stage			< 0.001
I–II	9 (32.1)	18 (85.7)	
III–IV	19 (67.9)	3 (14.3)	
WHO performance status			0.727
0–1	19 (67.9)	18 (85.7)	
>1	7 (25.0)	3 (14.3)	
NA	2 (7.1)		
Extranodal effusion			0.467
0–1	22 (78.6)	18 (85.7)	
>1	6 (21.4)	3 (14.3)	
IPI			0.111
0–2	14 (50.0)	17 (81.0)	
3–5	12 (42.9)	4 (19.0)	
NA	2 (7.1)		

**Table 2:** Antibodies used in immunostaining methods and Western blotting analysis. The dilute for all antibodies used in Western blotting analysis was 5% BSA. Trx: thioredoxin; TXNDC: thioredoxin domain-containing protein; 8-OHdG: 8-hydroxy-2′-deoxyguanosine; RT: room temperature; m/w: microwaved.

		Immunohistochemistry			Western blotting
Antibody	Source of antibody	Concentration	Incubation	m/w	Dilution
Trx	#2429, Thioredoxin 1 (C63C6) Cell Signaling Technology Inc., Danvers, MA, USA	1 : 400	1 h RT	Tris-EDTA pH 9, 10 min	—
TXNDC2	NBP1-80724 Novus Biologicals, Littleton, CO, USA	1 : 100	1 h RT	Tris-EDTA pH 9, 10 min	1 : 500
TXNDC3	NBP1-85616 Novus Biologicals, Littleton, CO, USA	1 : 200	1 h RT	Tris-EDTA pH 9, 15 min	1 : 2500
TXNDC6	NBP2-15152 Novus Biologicals, Littleton, CO, USA	1 : 250	1 h RT	Tris-EDTA pH 9, 10 min	1 : 1000
8-OHdG	MOG-100P, anti-8-hydroxy-2′-deoxyguanosine (N45.1) JaICA, Fukuroi, Japan	1 : 70	1 h RT	Citrate buffer pH 6, 15 min	—
Beta-actin	NB600-501 Novus Biologicals, Cambridge, UK	—	—	—	1 : 20 000
PCNA	#2586, Cell signaling Technology, Danvers, Ma, USA				1 : 2000
Anti-rabbit HRP-linked	Cell Signaling Technology, Danvers, MA, USA	—	—	—	1 : 3000
Anti-mouse HRP-linked	Cell Signaling Technology, Danvers, MA, USA	—	—	—	1 : 5000

## Data Availability

The data used in this study is available from the corresponding author upon request.
